# Fusing Artificial Cell Compartments and Lipid Domains Using Optical Traps: A Tool to Modulate Membrane Composition and Phase Behaviour

**DOI:** 10.3390/mi11040388

**Published:** 2020-04-07

**Authors:** Adithya Vivek, Guido Bolognesi, Yuval Elani

**Affiliations:** 1 Department of Chemistry, Imperial College London, London W12 0BZ, UK; adithya.vivek17@imperial.ac.uk; 2 Department of Chemical Engineering, Loughborough University, Leicestershire LE11 3TU, UK; G.Bolognesi@lboro.ac.uk; 3 Department of Chemical Engineering, Imperial College London, London SW7 2AZ, UK; 4 fabriCELL, Imperial College London, London SW7 2AZ, UK

**Keywords:** optical traps, vesicles, artificial cells, membrane biophysics, phase separation, membranes

## Abstract

New technologies for manipulating biomembranes have vast potential to aid the understanding of biological phenomena, and as tools to sculpt novel artificial cell architectures for synthetic biology. The manipulation and fusion of vesicles using optical traps is amongst the most promising due to the level of spatiotemporal control it affords. Herein, we conduct a suite of feasibility studies to show the potential of optical trapping technologies to (i) modulate the lipid composition of a vesicle by delivering new membrane material through fusion events and (ii) manipulate and controllably fuse coexisting membrane domains for the first time. We also outline some noteworthy morphologies and transitions that the vesicle undergoes during fusion, which gives us insight into the mechanisms at play. These results will guide future exploitation of laser-assisted membrane manipulation methods and feed into a technology roadmap for this emerging technology.

## 1. Introduction

Cell membranes are universal biological motifs that define cellular boundaries. They allow for cells to compartmentalise content, control the influx/efflux of materials, and they act as a surface on which biochemical reactions occur. Synthetic versions of cell membranes are not only used to shed light on fundamental principles underlying the function of membranes away from the complex cellular environment, but increasingly as components of artificial cells in the field of bottom-up synthetic biology [1,2,3].

The fusion of two cell membranes is a ubiquitous event in biological systems. Cell fusion is integral to a host of cellular processes, including endocytosis, exocytosis, cell division, cell-cell communication, and neurotransmission [4]. Bioengineers are increasingly exploiting this phenomenon, with synthetic membrane fusion events playing key roles in liposomal drug delivery [5], cell-like microreactors [6,7], cell transfection [8], the creation of cell hybrids for vaccine generation [9], and for therapeutic applications [10,11]. Membrane fusion is also being deployed in synthetic biology, to initiate protein synthesis in artificial cells through the delivery of genetic material [12] and in the creation of artificial cells that are capable of growing over time by subsuming new membrane material [13].

Fusion techniques generally fall into two camps. The first is bulk fusion, where the inherent biophysical properties of the membranes lead to stochastic fusion events in a vesicle population (e.g., through charge [14], membrane-bound DNA nanotechnology [15], and membrane mechanical properties [16]). The second is externally triggered fusion, where defined vesicles are brought into contact and fusion is initiated through applied forces (e.g., using electric fields [17,18] or lasers [12,19]). The attraction of the latter is the full level of spatiotemporal control of the fusion process that is available. Not only can a user define which vesicle will fuse with which, by the timing, spatial arrangement, and the number of fusion events can also be controlled.

There have been several recent studies on techniques to fuse Giant Unilamellar Vesicles (GUVs; enclosed cell-sized bilayer spheres) while using optical traps and membrane-bound gold nanoparticles (AuNP) [12,19,20]. The underlying mechanism behind this process is AuNP absorbance at the laser focus, followed by heat dissipation, local temperature elevation (>100 °C) in a region of characteristic size that is equal to the NP diameter [21], membrane expansion, and then fusion [19,20]. Similar optical tweezing technologies have recently been used to controllably manipulate co-existing membrane domains (analogues of cellular lipid rafts) within the membrane [22].

Several applications of laser-assisted vesicle fusion technologies have been demonstrated, including for the initiation of chemical reactions in vesicle microreactors [12], the creation of hybrid cells [20], and sequential dilution of encapsulated aqueous material [12]. However, the full capabilities of this technology have not been fully explored, and the potential for it to be used to modulate the composition and spatial arrangements of the membrane itself (and not simply mixing the encapsulated cargo) is largely an unchartered frontier.

Herein, we address this shortcoming through a series of proof-of-principle experiments on GUVs. Firstly, we show for the first time that laser-mediated vesicle fusion can be used to dynamically modulate the membrane composition and phase state by introducing new membrane material. Secondly, we use this technology to controllably fuse individual coexisting membrane domains with one another. Finally, we present a phenomenological account of some striking vesicle morphologies that the vesicle adopts during fusion, which have the potential to inform the underlying biophysical rules governing the fusion process. This work acts a roadmap for future work by highlighting the potential of optical trapping in membrane manipulation for a range of soft-matter biotechnology applications.

## 2. Results and Discussion

### 2.1. Dynamic Modulation of Lipid Composition through Vesicle Fusion

**Figure 1:**
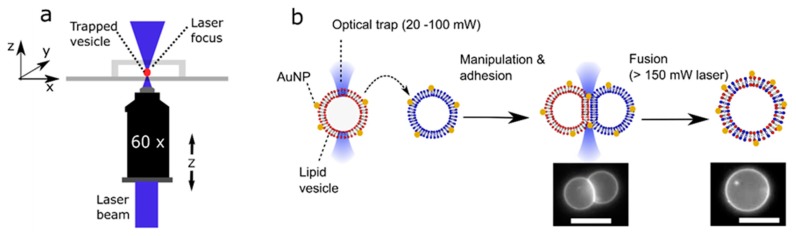
Schematic of the processes involved in laser-assisted vesicle adhesion and fusion. (**a**) Experimental setup. By moving a motorized stage and the objective itself, a vesicle could be manipulated in the x, y, and z direction relative to its surroundings. (**b**) Gold nanoparticles (AuNP) labelled vesicles of defined composition are brought together using an optical trap where they adhere due to the presence of NaCl in the external solution. Focussing the laser at the adhesion patch is used to initiate fusion, forming a unified structure with lipids originating from two previously distinct vesicles. Images of fluorescently labelled Giant Unilamellar Vesicles (GUVs) pre- and post-fusion, as shown. Scale bar = 5 µm.

The use of optical traps to select, manipulate, and fuse user-defined vesicles with another opens the possibility of dynamically and controllably modulating membrane composition by introducing new lipid cargo. Although fusion between Small Unilamellar Vesicles (LUVs; c. 100 nm diameter) and GUVs has been used to introduce protein machinery into membranes [23] and induce GUV growth [13], this method cannot be used to controllably alter the lipid composition due to the lack of control of the number of fusion events. In principle, laser-assisted GUV fusion offers precise spatial and temporal control which bypasses this limitation. The experimental setup (Figure 1) consists of a single-beam optical trap that is built into an inverted epi-fluorescence microscope system allowing for the user to select AuNP labelled vesicles, bringing them into contact to form an adhesion patch (laser: 20–100 mW at trap), and induce fusion by increasing the laser power (>150 mW at trap). The adhesion patch is formed due to the presence of 0.2 mM NaCl in the external solution which screens out electrostatic repulsion between the vesicles, allowing attractive forces to dominate [12]. The fusion event is triggered by the interaction of the continuous laser beam with the nanoparticles sitting in the adhesion patch.

What enabled the manipulation of the lipid vesicles using an optical trap was the difference in molecular composition and, hence, refractive index between the vesicle interior (0.75 M sucrose) and exterior (0.35 M glucose, 0.2 M NaCl). This refractive index mismatch was enough to facilitate trapping and manipulation at laser powers above 20 mW at the trap.

We track changes in lipid composition post-fusion through the phase state of the membrane. Membranes can exist in a fully liquid phase (Lα), gel phase (Lβ) or have co-existing phase-separated domains [24]. In our experiments, there are either gel/liquid coexisting domains, or liquid-ordered/liquid-disordered (L_o_/L_d_) coexisting domains. The phase state is dependent on the precise lipid composition of the membrane [24,25], and it can be visualised using fluorescence microscopy through the incorporation of fluorescently labelled lipids (Rh-PE), which preferentially partitions into the disordered phases.

**Figure 2:**
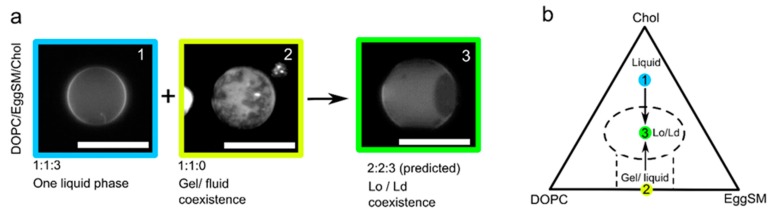
Changing membrane phase state through delivery of new lipid material. (**a**) Two giant vesicles of different compositions and phase states are fused together using an optical trap. Adding a fully fluid vesicle to one showing gel/fluid domain coexistence (irregularly shaped domains) yielded a vesicle exhibiting liquid-ordered/liquid disordered domain coexistence (spherical domains). Scale bar = 5 µm. (**b**) A schematic demonstrating adding lipid material from vesicle (1) to vesicle (2) leading to a new vesicle (3) occupying a different part of the phase diagram. Note: this is not an empirical phase diagram; it is an approximate one used for illustrative purposes only.

In these experiments, we use ternary lipid mixtures. The lipids we used were: DOPC, which promotes the formation of a liquid phase (L_α_) at room temperature; EggSM, which promotes the formation of a gel phase (L_β_) at room temperature; and cholesterol, which promotes the formation of the liquid-ordered (L_o_) phase in ternary mixtures. Using the laser-assisted fusion technique, we were able to move a vesicle from a gel/liquid coexisting regime to an L_o_/L_d_ one, by delivering cholesterol-rich membrane material (Figure 2). The ternary lipid composition used for this experiment was DOPC/EggSM/Chol with the constituent lipids that were present in different ratios. Using iso-osmotic conditions, we fused two vesicles of c. 5 µm diameter, one of which was a 1:1:0 GUV (gel/liquid; small speckled non-spherical domains) with a 1:1:3 GUV (L_α_; no domains, uniform fluorescence). This changed the membrane composition to lie in the L_o_/L_d_ region of the phase diagram (large spherical domains) [24]. Assuming that the vesicles were identical in size, the final vesicle is expected to have a DOPC/EggSM/Chol 2:2:3 composition.

### 2.2. Manipulation and Fusion of Defined Coexisting Domains

**Figure 3:**
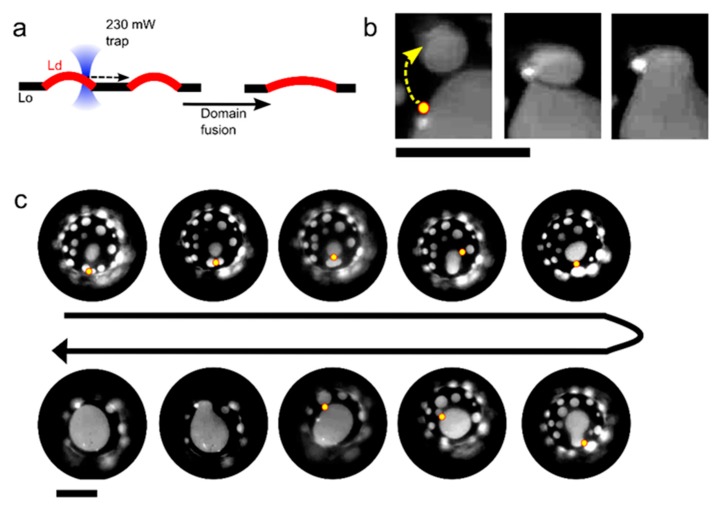
Controlled manipulation of membrane domains using optical traps. (**a**) Schematic showing manipulation of liquid-disordered (L_d_) domains within a liquid-ordered (L_o_) matrix to induce domain fusion. (**b**) Fluorescence image of a portion of a vesicle membrane showing an L_d_ domain being dragged by an optical trap (yellow dot and arrow) towards an adjacent domain to induce fusion. Lipid composition: DOPC/EggSM/Chol 1:1:1. (**c**) Time-course image showing eight sequential fusion events that were user-defined through domain manipulation with an optical trap (yellow circle). Scale bars = 5 µm.

In addition to manoeuvring and fusing membrane compartments with each other, we demonstrate that individual coexisting domains within a lipid membrane can be merged with spatiotemporal control. The ability to manipulate domains using optical forces has previously been shown [22] but using an optical trap to controllably fuse domains together has not. In a previous study [22], we have shown how the difference in both membrane refractive index and thickness between the L_o_ and L_d_ phase results in an optical gradient force that enables domain trapping and manipulation via a single trap. An approximated expression of the optical trapping force that was exerted by the laser on a trapped domain was also derived [22]. In the experiments below, we show that we can drag L_d_ domains across the membrane surface, and bring them in contact with adjacent domains to initiate fusion, by taking a DOPC/EggSM/Chol 1:1:1 vesicle and focussing the laser (0.23 W at trap) at the L_o_/L_d_ interface (Figure 3; Video S1). We were able to sequentially fuse nine isolated domains to yield a domain of an incrementally increasing size using this method. We note that, with this composition, domains did not naturally coalesce within the timescales of the experiment, due them having a ‘bulged’ morphology, where they protrude out the vesicle body. Domain bulging means that the total interface length of the domain boundary is reduced, in turn reducing the total line tension, which is energetically favourable [24,26,27]. Ordinarily, domains would collide and coalesce, eventually growing into one large domain to reduce the total interface length. However, the bulged geometry creates repulsion between domains, allowing for multiple domains to be stabilized in a kinetically trapped regime. In our experiments, the repulsion between domains is overcome while using the optical trap through two processes operating in tandem. The first is that the traps are used to bring the domains together, overcoming the inter-domain repulsion. The second is through the heat emanating from the laser focus and the resulting temperature increase (maximum increase estimated to be 4–6 °C while using the laser powers used) [12]. This reduces the line tension, which in turn reduces the extent of domain bulging and the lowering of the inter-domain repulsion.

### 2.3. Morphological Transitions

Vesicles tended to go through a series of common morphological transitions during the fusion process, which can give us insight into the underlying biophysical processes at play. The most common of these are described below, together with speculation regarding their origins.

**Figure 4:**
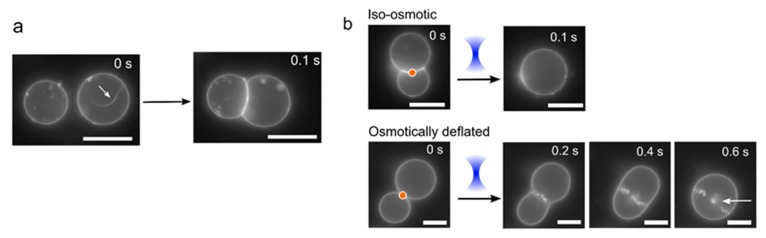
Vesicle morphology changes under different osmotic conditions. (**a**) If vesicles have excess membrane area and low tension, they may contain tubules (white arrow). Upon adhesion, the vesicle deform to a non-spherical geometry, which increases its tension and leads to a retraction of the tubule into the main vesicle body. (**b**) Images showing post-fusion morphologies under iso-osmotic (0.75 M sucrose internally; 0.35 M glucose, 0.2 M NaCl externally) and osmotically deflated (0.75 M sucrose internally; 0.4 M glucose, 0.2 M NaCl externally; low tension) conditions. In the former, a clean-merge is seen; in the latter the vesicle slowly relaxes to a spherical shape and many smaller internal vesicles are produced and retained inside (white arrow). Orange dot corresponds to area of laser focus. Scale bars = 5 µm.

The first relates to a morphological change during vesicle adhesion, where the vesicles ‘zip-up’ to form a straight interface membrane. When vesicles are generated, there is a large vesicle-to-vesicle variation in the surface tensions, depending on how much lipid material ends up in the final structure. On occasions where there is excess membrane surface area, protruding tubules are observed. When such vesicles are brought into contact with other vesicles, the tubules disappear, and they are quickly (<100 ms) subsumed into the main membrane body after adhesion (Figure 4a; Video S2). This is because, during adhesion, the total vesicle surface area increases as it deforms away from a spherical geometry. This leads to a corresponding increase in vesicle tension, as the encapsulated volume remains the same, leading to a retraction of the tubule. Assuming identical vesicle size, the ratio of the vesicle surface area increase due to membrane adhesion over the original vesicle area is given by

(1) $$f\left(\vartheta \right)=\frac{3-\cos\vartheta }{2^{2/3}\;{\left(1+\cos\vartheta \right)}^{1/3}\;{\left(2-\cos\vartheta \right)}^{2/3}}-1$$

In our experiments, the contact angle ϑ, defined as the arcsine of the adhesion patch diameter normalised with respect to the vesicle diameter, can vary between a few degrees up to c. 40°, thus resulting in relative increases of vesicle surface area of c. 1%. With a typical elastic expansion area modulus of order 200 mJ/m^2^, the vesicle membrane tension can increase by up to few tens of µN/m, which can be enough to destabilise the nanotubules that protrude from the vesicle surface.

After adhesion, the vesicles were fused with one another by exposing the vesicle/vesicle interface to a continuous laser (>150 mW). Different behaviours were observed in iso-osmotic and osmotically deflated vesicles (Figure 4b). In iso-osmotic conditions (0.75 M sucrose internally, 0.35 M glucose 0.2 M NaCl externally), clean fusion events occurred 82% of the time (n = 50) with a single post-fusion vesicle formed almost instantaneously (<100 ms) after exposure to the laser (Video S3). In contrast, when the vesicles were osmotically imbalanced (0.75 M sucrose internally, 0.4 M glucose and 0.2 M NaCl externally) and, hence, had lower membrane tensions, disorderly fusion morphologies were always observed (Figure 4b, Video S4). These took the form of pearling instabilities, where tens of smaller vesicles are generated (and retained inside) the post-fusion vesicle.

One possible explanation for this is the creation of high energy exposed bilayers following laser exposure. Because of the low-tension and flaccid nature of the membranes, these are free to deform and reseal quickly with membrane segments that are in close proximity. This would lead to the formation of further exposed membranes in an iterative process that results in the generation of several internal vesicles. In principle, the internal membrane structures may be connected by small membrane tubules that are below the resolution of the microscope. Furthermore, the merging of two vesicles of identical size results in a post-fusion vesicle whose membrane area is c. 20% less than the combined membrane area of the two original vesicles due to the conservation of the encapsulated volume. This area reduction combined with the relatively large membrane excess area of the two deflated merging vesicles might promote the formation of membrane structures other than a single larger post-fusion vesicle, such as smaller vesicles and nanotubules.

**Figure 5:**
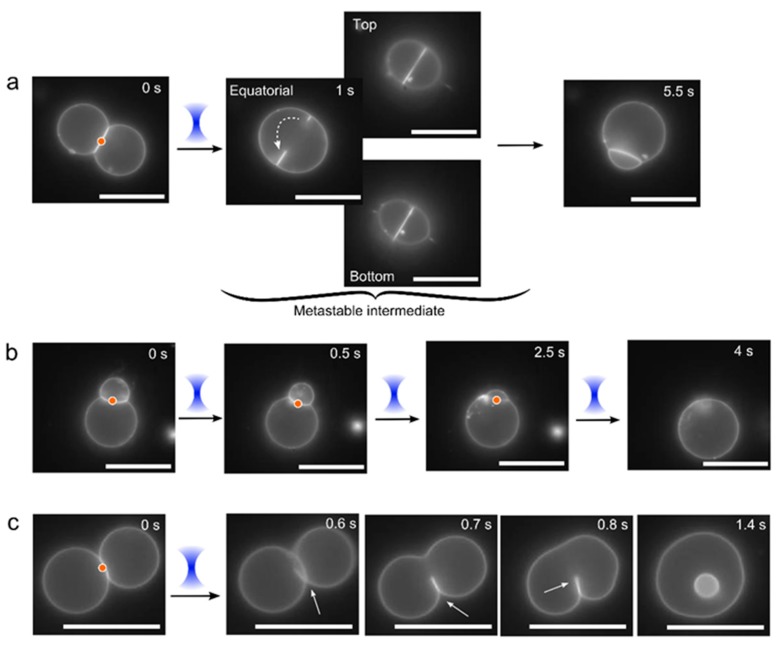
Post-fusion morphologies and intermediate structures. Under osmotically balanced conditions (0.75 M sucrose internally; 0.35 M glucose and 0.2 M NaCl externally) several alternative non-clean fusion events were occasionally observed after application of the laser (red dot and blue cone), including: (**a**) A meta-stable pore of c. 3 µm diameter in the bilayer partition, which after c. 5s rearranged (dotted arrow) to reform the interface membrane. (**b**) Quick opening and closing of a pore upon continuous laser illumination, after each event a portion of the membrane of one vesicle was subsumed into the other, with the formation of a single vesicle at the end. (**c**) An exposed pendant interface membrane (white arrow), which rearranged and reconnected with itself to yield in an inner vesicle. Scale bar = 5 µm.

However, several interesting ‘disorderly’ fusion events were also occasionally observed in iso-osmotic conditions (Figure 5). There were instances when intermediate bodies formed that were stable for c. 4 s before collapsing to generate internal membrane partitions (Figure 5a; Video S5). The intermediate structures appeared to be membranes that possessed a visible hole >3 µm in diameter. The hole resealed when the open membrane edges travelled along the surface of the vesicle, minimising the size of the pore, until the two edges met and then formed a complete internal membrane. In other instances, if the laser was continuously left on, multiple sequential opening and re-sealing events were seen. In this case, one of the vesicles would get smaller over time, until only a single unified vesicle was left (Figure 5b, Video S6). Finally, there were occasions where visible membrane pores would appear, followed by membrane rearrangement in order to yield a single internal vesicle encapsulated in a larger one (Figure 5c, Video S7).

## 3. Materials and Methods

### 3.1. Optical Trapping and Microscopy Setup

An inverted epi-fluorescence dual-carousel microscope (Nikon TE2000-U, Nikon Instruments, Tokyo, Japan) combined with an optical trapping system was used for imaging and optical manipulation, as described previously [12,28]. Briefly, the setup involves a conventional single beam optical trap that was based on a linearly polarised beam from an Ytterbium fibre laser source (20 W at 1070 nm; IPG Photonics, Europe). A bespoke 30 mm cage optic and filter cube mount was machined and replaced the upper carousel of the microscope. The laser collimator head was mounted in a cage plate and the beam was expanded to fill the back aperture of a 60 × 1.4 NA oil-immersion objective with a pair of opposing plano-convex lenses in a Keplerian arrangement along a cage optic rail. The IR dichroic mirror and IR filter (Chroma, Bellows Falls, VT, USA) were fitted in a filter cube that was mounted in the bespoke mount. The laser power at the back aperture was measured to be 9.9 ± 0.2% of the nominal laser launch power. The laser focal point was aligned to coincide with the object plane and objects were manipulated by the translation of the motorised microscope XY stage and the objective along the *z*-axis. The sample was imaged while using the CCD camera (ORCA-ER Hamamatsu, Shizuoka, Japan). A TRITC Nikon filter cube and a mercury-fibre illuminator (Nikon Intensilight CHGFIE, Tokyo, Japan) were used for imaging the vesicles in fluorescence mode (50 ms exposure). Image acquisition was controlled using a customised Labview (National Instruments Corp, TX, USA) interface. We note that, in all our experiments, we manipulated vesicles one at a time using a single laser. Future work concerning more advanced applications in which simultaneous trapping of multiple objects are needed will require modifications to the setup, for example, through the integration of holographic optical tweezers or time-sharing optical traps systems.

### 3.2. Vesicle Generation

All of the lipids were purchased from Avanti Polar Lipids (Alabaster, AL, USA) and, unless otherwise specified, reagents purchased from Sigma Aldrich (Gillingham, UK). Unless otherwise stated, all of the vesicles were composed of 1-palmitoyl-2-oleoyl-glycero-3-phosphocholine (POPC). For domain experiments, other lipids used were 1,2-dioleoyl-sn-glycero-3-phosphocholine (DOPC), Egg Sphingomyelin (EggSM), and Cholesterol (Chol). GUVs were formed via electroformation in a sucrose solution in DI water (0.75 M, unless otherwise specified in the text). Lipid solutions containing various ratios of different lipids (given in the text) were prepared together with 1 mol% fluorescent lipid 1,2-dioleoyl-sn-glycero-3-phosphoethanolamine-N-(lissamine rhodamine B sulfonyl) (RhPE). These were prepared by dissolving appropriate amounts of lipid in chloroform to yield a 1 mg mL^−1^ solution. 40 µL of this solution was then spread evenly on an indium tin oxide (ITO) slide, and a lipid film was deposited as the chloroform evaporated. The slide was placed in a desiccator for a minimum of 30 min to remove residual chloroform. A 5 mm thick polydimethylsiloxane (PDMS) spacer with a central cut-out was sandwiched in between two ITO slides, one of which contained the lipid film, with the conductive sides facing each other. This chamber was held together with clips, and it was filled with the 0.75 M sucrose solution. An alternating electric field (1 V, 10 Hz) was applied across the ITO plates while using a function generator (Aim-TTi, TG315, Huntingdon, UK). The sample was left to electroform in a 60° oven to ensure that the lipid was in the fluid phase. After two hours, the electric field was changed to 1 V, 2 Hz for a further hour, and the resulting vesicles collected.

### 3.3. Fusion Experiments

For vesicles fusion, there needed to be AuNPs present at the vesicle-vesicle interface. This was achieved by functionalizing the vesicles by adding 2 wt.% 16:0 Biotinyl Cap PE to the initial lipid film. After vesicle formation, 150 nm streptavidin-coated AuNPs (Nanopartz, CO, USA; product C11-150-TS-50; 2.5 mg·mL^−1^) were added to the vesicles 1:9, with the sample being vortexed for 30 min to drive conjugation. For the fusion experiments, the vesicles were deposited on a coverslip coated with a BSA monolayer to prevent interactions between the glass substrate and the vesicle membrane. The coating was applied by depositing 300 µL of 1% BSA in DI water on a coverslip and leaving it to evaporate in a 60 °C oven, leaving behind a protein film. The film was subsequently rinsed with DI water and dried under a nitrogen stream. The vesicle assembly chambers were prepared by placing a 1 mm thick PDMS sheet with a 10 mm diameter hole on the coverslip.

For the fusion experiments, unless otherwise stated, all of the experiments were conducted under iso-osmotic conditions. A sample of vesicles with 0.75 M sucrose internally, and 0.35 M glucose, 0.2 M NaCl externally were created by diluting the vesicles following electroformation with appropriate volumes of NaCl and glucose solutions, all in DI water. The sample was then mixed by pipette aspiration, the chamber sealed with a second coverslip, and then placed on the optical trapping setup. Individual vesicles were trapped by switching the laser on (20 mW–100 mW at trap), and were moved in three-dimensional (3-D) relative to the sample by moving the microscope stage (x,y) and changing the focus of the objective (z). These relatively low powers were needed to avoid unintended fusion. Once the vesicles were brought into contact with one another to form an adhesion patch, fusion was initialised by applying a laser power of >150 mW (at trap) at the membrane patch.

## 4. Conclusions

We have conducted a series of feasibility experiments to demonstrate the potential of laser-assisted membrane manipulation. Firstly, we show that optical trapping technologies can be used to dynamically move across a membrane phase diagram by changing the composition of a bilayer through the delivery of new membrane material. This is powerful, as it paves the way for systematic changes to the lipid composition of the membrane while still retaining the encapsulated cargo. It also enables the study of non-equilibrium dynamic processes, such as the kinetics of membrane rearrangement after perturbations in lipid compositions are imposed. Unlike alternative methods (e.g., using reagents that remove specific membrane molecular constituents) [29], this is a generic method, which, in principle, allows for the introduction of any material (lipid, cholesterol, protein, etc.) embedded in vesicles, as well as the controlled addition of non-biological material (e.g., block copolymers, dendrimers etc.) in controlled quantities. Similar approaches based on electrofusion have been shown to bypass issues that were associated with batch variation of vesicle lipid composition by changing the lipid composition post-vesicle generation [18]. Despite the laser-induced fusion, NP-labelled membranes may result in relatively larger temperature (>100 °C) that could compromise the functionality of biological material; such a temperature increase is localised in a small region of a size that is comparable to the NP diameter (100 nm). Since outside this region the temperature decreases as the inverse of the distance, it is expected that the vast majority of the cargo remains unaffected.

Secondly, we show the controlled fusion of coexisting domains in a vesicle. This can unlock applications for controlled chemical reactions on a membrane surface, for example, if reactive species can be bound to defined domains, and it can be deployed in future in studies that concern the biophysics of domain coalescence.

Finally, the fine spatiotemporal control that is associated with the use of laser has meant that we were able to observe a repertoire of membrane transformations, including: (i) the formation of large open membrane intermediate structures that appear to be metastable; (ii) the opening/resealing of transient pores (<1 s); and, (iii) the generation of internal daughter vesicles post-fusion. Many of these could be repurposed in a synthetic biology context. When coupled with microfluidic [30] and related technologies [31,32], it could be used to sculpt new cell-mimetic motifs (e.g., synthetic nuclei), replicate cellular processes (e.g., endocytosis), and gain insight into cell membrane biophysics in a model environment. Taken together, these results serve as a feasibility roadmap to guide future detailed studies and exploitation of laser-assisted membrane manipulation technologies.

## Supplementary Materials

The following are available online at https://www.mdpi.com/2072-666X/11/4/388/s1: Video S1: Controlled optical manipulation and fusion of membrane domains. Video S2: Post-adhesion vesicle deformation and retraction of the tubule. Video S3: Clean vesicle fusion under iso-osmotic conditions. Video S4: Disorderly vesicle fusion under osmotically deflated conditions. Video S5: Transient visible membrane pores post-fusion. Video S6: Multiple pore opening and closing events upon laser exposure. Video S7: Post-fusion generation of a single internal daughter vesicle.
